# Repurposing existing medications as cancer therapy: design and feasibility of a randomized pilot investigating propranolol administration in patients receiving hematopoietic cell transplantation

**DOI:** 10.1186/s12885-018-4509-0

**Published:** 2018-05-24

**Authors:** Jennifer M. Knight, Stephanie A. Kerswill, Parameswaran Hari, Steve W. Cole, Brent R. Logan, Anita D’Souza, Nirav N. Shah, Mary M. Horowitz, Melinda R. Stolley, Erica K. Sloan, Karen E. Giles, Erin S. Costanzo, Mehdi Hamadani, Saurabh Chhabra, Binod Dhakal, J. Douglas Rizzo

**Affiliations:** 1 Departments of Psychiatry, Medicine, and Microbiology & Immunology, Medical College of Wisconsin, 8701 Watertown Plank Road, Milwaukee, WI 53226 USA; 2 Medical College of Wisconsin, Milwaukee, WI USA; 3 Division of Hematology/Oncology, Department of Medicine, Medical College of Wisconsin, 8701 Watertown Plank Road, Milwaukee, WI 53226 USA; 4 Department of Medicine, Division of Hematology-Oncology, and Department of Psychiatry and Biobehavioral Sciences, David Geffen School of Medicine at UCLA, Los Angeles, CA USA; 5 Center for International Blood and Marrow Transplant Research; Medical College of Wisconsin, Milwaukee, WI USA; 6 Division of Biostatistics, Institute for Health & Society, Medical College of Wisconsin, Milwaukee, USA; 7 Department of Medicine, Medical College of Wisconsin, Milwaukee, WI USA; 8 Monash Institute of Pharmaceutical Sciences, Monash University, Clayton, VIC Australia; 9 Cousins Center for Psychoneuroimmunology, Semel Institute for Neuroscience and Human Behavior, Jonsson Comprehensive Cancer Center, and UCLA AIDS Institute, UCLA, Los Angeles, CA USA; 10 Division of Cancer Surgery, Peter MacCallum Cancer Centre, Victorian Comprehensive Cancer Centre, Melbourne, VIC Australia; 11 Carbone Cancer Center and Department of Psychiatry, University of Wisconsin-Madison, Madison, WI USA

**Keywords:** ß-blocker, Propranolol, Feasibility, Repurposed drugs, Multiple myeloma, Hematopoietic cell transplantation

## Abstract

**Background:**

Repurposing existing medications for antineoplastic purposes can provide a safe, cost-effective, and efficacious means to further augment available cancer care. Clinical and preclinical studies suggest a role for the ß-adrenergic antagonist (ß-blocker) propranolol in reducing rates of tumor progression in both solid and hematologic malignancies. In patients undergoing hematopoietic cell transplantation (HCT), the peri-transplant period is a time of increased activity of the ß-adrenergically-mediated stress response.

**Methods:**

We conducted a proof-of-concept randomized controlled pilot study assessing the feasibility of propranolol administration to patients between ages 18–75 who received an autologous HCT for multiple myeloma. Feasibility was assessed by enrollment rate, tolerability, adherence, and retention.

**Results:**

One hundred fifty-four patients underwent screening; 31 (20%) enrolled in other oncology trials that precluded dual trial enrollment and 9 (6%) declined to enroll in the current trial. Eighty-nine (58%) did not meet eligibility requirements and 25 (16%) were eligible; of the remaining eligible patients, all were successfully enrolled and randomized. The most common reasons for ineligibility were current ß-blocker use, age, logistics, and medical contraindications. 92% of treatment arm patients tolerated and remained on propranolol for the study duration; 1 patient discontinued due to hypotension. Adherence rate in assessable patients (*n* = 10) was 94%. Study retention was 100%.

**Conclusions:**

Findings show that it is feasible to recruit and treat multiple myeloma patients with propranolol during HCT, with the greatest obstacle being other competing oncology trials. These data support further studies examining propranolol and other potentially repurposed drugs in oncology populations.

**Trial registration:**

This randomized controlled trial was registered at clinicaltrials.gov with the identifier NCT02420223 on April 17, 2015.

## Background

Cancer treatment is expensive. The rising cost of pharmacological advancements in cancer therapeutics is unlikely to be sustainable with current healthcare budgets [1]. In 2013, $91 billion was spent on oncology drugs alone [2], with the median cost of cancer drugs growing from <$100 to ~$10,000 per month over the last 20–30 years [3, 4]. Despite advances in targeted immunotherapies in cancer, these agents have had modest impact on event-free and overall survival [5]. Though current promising immunotherapy trials are in process [6, 7], it remains unknown whether or how these treatments might be limited by adverse effects. Emerging evidence suggests a biologically heterogeneous cancer milieu, necessitating the need for combination treatments [5, 8–10]. A variety of existing drugs used to treat non-cancer conditions may be efficacious in the treatment of cancer [11]. Repurposing existing medications for cancer provides a rational, evidence-based, and cost-effective approach to contribute solutions to these challenges; studying new indications for old drugs is far less expensive than developing new drugs, and many of these drugs are available as low cost generics [1]. However, much remains unknown about trial feasibility and efficacy of repurposed drugs in a cancer context.

The non-selective ß-adrenergic receptor (ßAR) antagonist (ß-blocker) propranolol is a commonly administered drug with promising antineoplastic properties. Its use as adjunctive therapy in cancer is supported by reports that propranolol diminishes stress-mediated tumor progression in animals [12–14] and is associated with reduced rates of cancer progression in retrospective human studies of both solid and hematologic malignancies [15–20]. Stress-associated factors are predictive of adverse cancer outcomes, including survival [21, 22]. Propranolol is the most studied nonselective β-blocker [23]. This, along with its tolerability, low cost, and efficacy in vitro in preventing tumor progression [24–26] make it an excellent candidate for cancer repurposing in humans. Though current trials are underway investigating ß-blocker use in cancer [27], there are limited published prospective human clinical studies. Two recently published studies evaluated its use in the peri-operative period; one evaluated propranolol at a consistently lower dose along with a cyclooxygenase− 2 inhibitor among breast cancer patients [28] and another assessed propranolol use in ovarian cancer patients [29]. A third non-randomized study showed propranolol protects patients with thick cutaneous melanoma from disease recurrence [30].

Hematopoietic cell transplantation (HCT) is an increasingly used treatment modality [31] whose course and outcomes could significantly benefit from adjunctive adrenergic blockade for several reasons. First, despite improved outcomes over the past 30 years, HCT is physically and psychologically arduous with notable morbidity and mortality [32]. Next, stress levels are high after transplant. In one study, 55% of individuals undergoing HCT endorsed elevated levels of anxiety and/or depression pre-transplant [33]. Another study shows rates of post-traumatic stress disorder and depression at 28 and 43%, respectively, in the months following HCT [34]. Stress peaks in the immediate peri-HCT period, gradually improving over time [35, 36]. This may be clinically relevant, as stress-associated factors are related to adverse outcomes following HCT [37–40]. Evidence suggests that biobehavioral factors – including the physiologic stress response – may significantly affect HCT outcomes, including relapse and survival [32, 38, 41, 42]. Consistent with the relapse observation, data from mouse models of cancer demonstrate that ß-blockade with propranolol prior to stress exposure blocks tumor-promoting signaling pathways in hematologic malignancies [14]. In sum, reducing the impact of the physiologic stress response may substantially increase the success of HCT.

Individuals with multiple myeloma comprise an HCT treatment group that may benefit from adjunctive propranolol administration. Multiple myeloma is the most common disease indication for HCT in the US, with most patients receiving autologous HCT. The latter is not curative, though it prolongs progression-free survival. Thus, patients face a chronic, recurrent condition and may be particularly distressed [43]. Recent retrospective evidence shows that ß-blocker use (for non-cancer reasons) is independently associated with better myeloma prognosis - including progression-free and overall survival - with no greater incidence of adverse effects [20]. In this study of 1971 multiple myeloma patients, myeloma-specific mortality rates at 5 years were 24% for patients taking only a ß-blocker (no other cardiac medications), 32% for a ß-blocker in addition to another cardiac drug, 41% for no cardiac drugs, and 50% for non-ß-blocker cardiac drugs*.* On a cellular level, propranolol has apoptotic and anti-proliferative effects on myeloma cells [44].

Feasibility trials are needed to unlock the potential for repurposing existing, affordable medications such as propranolol for anticancer purposes. It is important to understand the feasibility of recruiting and retaining patients in trials of repurposed drugs in the current oncology trial climate of targeted immunotherapy regimens; patients may be more interested in participating in trials of new agents and providers may have greater enthusiasm to refer patients to these trials. Further, it is necessary to understand how drug adherence, tolerability, and retention interface with complex cancer treatments such as HCT where nausea, hypotension, bradycardia, and volume depletion commonly occur and overlap with ß-blocker side effects. There are no published prospective randomized controlled trials (RCTs) reporting on use of ß-blockers alone in the cancer treatment setting, nor have any trials evaluated the use of ß-blockers in hematologic malignancies or HCT. Here, we report on a translational proof-of-concept pilot RCT designed for efficacy based on ß-adrenergically mediated gene expression outcomes that investigated the use of a ß-blocker in patients undergoing HCT. An important component of this trial was to determine acceptability of and adherence to a trial of a repurposed non-cancer drug in the setting of rigorous conventional cancer treatment.

## Methods

### Study design

This was a single site, proof-of-concept randomized controlled pilot study of propranolol administration to individuals undergoing first autologous HCT for multiple myeloma. We evaluated the clinical feasibility of using a ß-blocker in a cancer population undergoing an intensive conventional antineoplastic treatment regimen (HCT) as assessed by enrollment rate, tolerability, adherence, and retention. This was a secondary objective of the overall study. The primary objective of this study was to assess whether propranolol administration to myeloma patients undergoing HCT alters genome-wide transcriptional pathways involved in ß-adrenergic signaling and will be reported elsewhere.

### Eligibility criteria

The target patient population for this study was patients with multiple myeloma undergoing their first autologous HCT between 18 and 75 years of age. Additional eligibility criteria included not being on a contraindicated medication or ß-blocker within 3 weeks of study commencement; ≤1 year of initiation of systemic anti-myeloma therapy; no prior progression or relapse of myeloma prior to HCT; stable disease, partial response, or very good partial response at the time of HCT; able to receive melphalan 200 mg/m^2^ as a conditioning regimen; available hematopoietic cell graft with > 2.0 × 10^6^ CD34^+^ cells/kg available for transplant; Karnofsky Performance Score (KPS) of ≥80%. Exclusion criteria included previous intolerance to ß-blocker therapy, contraindications to ß-blocker therapy, and active, untreated depression.

### Enrollment and randomization

Patients were approached for this study by their primary transplant physician at a routine outpatient HCT clinic visit during the pre-transplant evaluation phase or stem cell mobilization (≥1 week prior to actual study commencement of Day − 7 with respect to day of transplant being Day 0; nomenclature throughout will continue as such). Per institutional practice, patients were informed of and offered all clinical trials for which they were eligible. Eligible patients willing to participate in the trial signed a Medical College of Wisconsin (MCW) institutional review board (IRB)-approved informed consent form. After consent was obtained, participants were randomized via permuted block assignment with random block sizes to receive propranolol or control (no additional intervention). The study was not blinded due to the objective nature of the study’s primary aim of gene expression profiling. Patients were enrolled at their consent visit, with study commencement at 7 days before transplant (Day − 7 ± 2 days), well after stem cell collection and granulocyte-colony stimulating factor administration for multiple myeloma patients at MCW*.* Intervention group patients were monitored on a weekly basis for 7 weeks (Table 1); 1 week pre-transplant through 6 weeks post-transplant) while control patients were monitored weekly for 5 weeks (1 week pre-transplant through 4 weeks post-transplant).

**Table 1:** Study assessment schedule

Study Assessment Time Point	Target Day
Baseline	Up to Day −7
Pre-Transplant	Day −2 ± 1 day
Day 0	Date of transplant
1 week	7 ± 2 days
2 weeks	14 ± 2 days
3 weeks	21 ± 3 days
4 weeks	28 ± 3 days
5 weeks	35 ± 3 days
6 weeks	42 ± 3 days
Next clinic appointment	~ 8 weeks (post-transplant)
14 weeks	100 ± 2 days

**Fig. 1:**
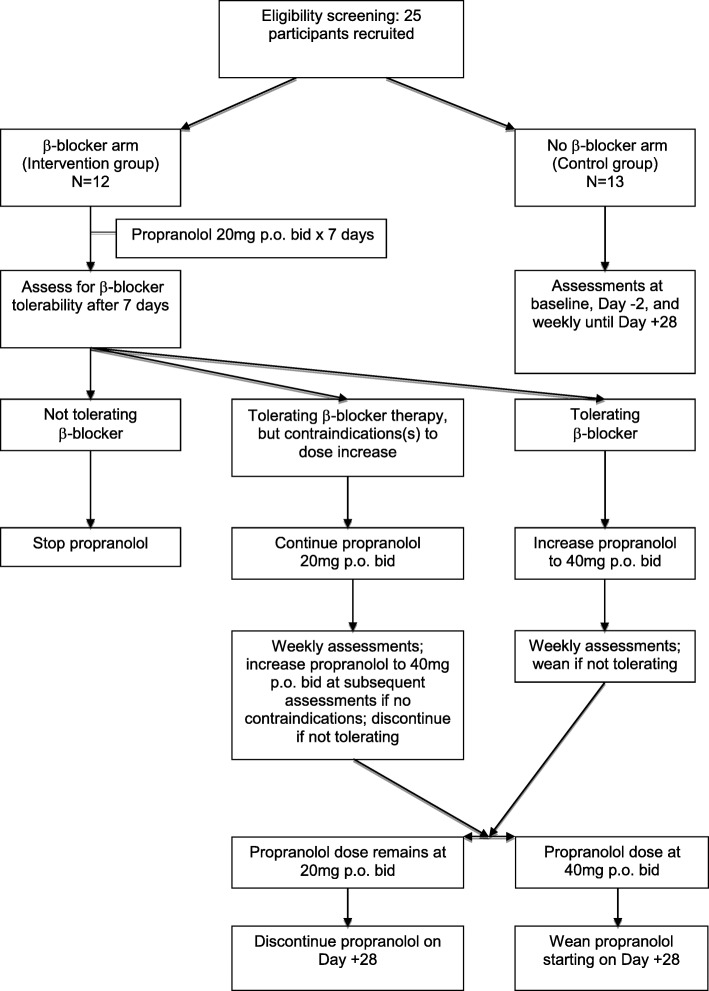
Treatment Schema

Intervention group participants were followed longer to allow for appropriate monitoring during propranolol weaning. Additional clinical information was collected for up to 100 days post-transplant as identified in section 2.5. See treatment schema described in Fig. 1 for full enrollment and drug dosing details.

### Intervention

#### ß-blocker timing and dosing

Participants began propranolol 20 mg orally twice daily (bid) at the time of study commencement (Day − 7 ± 2 days). Propranolol was taken twice daily until Day + 28 post-transplant, during a period of high psychological and physiological stress and inflammatory processes in patients undergoing autologous HCT [45]. Stress peaks in the immediate peri-HCT period and gradually improves over time, with a return to pre-transplant levels by about 1 year post-transplant [35, 36]. It follows that the first 30 days may also be the time period of highest ß-adrenergic signaling due to an increased stress response. Further, preclinical findings demonstrate that ß-blockade with propranolol 8 days prior to exogenous stress exposure is effective in blocking ß-adrenergic signaling at the tumor level [14]. Therefore, we ß-blocked patients during a physiological timeline in which ß-adrenergic gene expression is most likely to be affected.

The Principal Investigator (PI), treating HCT physician, and study coordinator assessed drug tolerability to adjust dosing after 1 week. If participants were tolerating propranolol without any side effects (see Study Monitoring section below for assessment details), the dose was increased to 40 mg bid after a week. If participants had noticeable but less severe side effects and were able to tolerate staying on propranolol, their dose was maintained at 20 mg bid. Study participants who remained at 20 mg orally bid continued on as study subjects.

Propranolol’s unique pharmacokinetic profile [46, 47] makes it difficult to calculate a predicted serum concentration. A mouse serum concentration target of 41 ng/mL (range 27–76 ng/mL) has been established to mitigate adverse ß-adrenergic effects on tumor progression in mice and theoretically correlates with target human serum concentration [48, 49]. This concentration was sustained at days 21 and 28 with 10 mg/kg/day dosing in a 28-day slow release pellet. For a 20 g mouse this equates to 0.2 mg/mouse/day.

We applied human dose-finding studies to determine the human equivalent dose (HED) needed to achieve said concentration. Animal to human dosing may be converted using the dose translation formula based on body surface area where HED (mg/kg) = animal dose (mg/kg) multiplied by Animal Km/Human Km (where animal Km = 3, human Km = 37) [50]. The HED is 56 mg/day for a 70 kg human. However, the 10 mg/kg/day dose used in mouse models is sustained release and is not a single-dose; therefore, with the sustained-release formulation the HED could be as low as 6 or 7 mg/day or 7 mg/day. The actual HED is likely near the midpoint of the low and high values calculated. Consequently, a goal dose of 20–40 mg of propranolol orally bid in humans is expected to achieve more than adequate serum concentrations as that demonstrated in mice to affect cancer progression.

#### ß-blocker weaning

Patients were weaned off propranolol for one of three reasons: 1) study completion, 2) intolerance secondary to side effects, or 3) onset of new medical symptoms rendering ß-blocker therapy contraindicated. For patients who were at 40 mg bid at the time of weaning, the dose was reduced to 20 mg bid for 1 week before being discontinued entirely. Patients who were treated with 20 mg bid at the time of weaning were discontinued immediately.

### Study monitoring

The study drug was dispensed by the cancer center pharmacy and compliance monitored by clinical research coordinators (CRCs). Tolerability was assessed clinically in both arms by the study CRC and PI on a weekly basis and once at the 1-week post-propranolol time point; subjects were questioned about side effects such as fatigue, dizziness, constipation, bradycardia, depression, insomnia, weakness, disorientation, nausea, diarrhea, hypersensitivity reactions, purpura, alopecia, and impotence. Blood pressure and heart rate were assessed during weekly appointments. Adverse events (AEs) were recorded in compliance with the National Cancer Institute’s Common Terminology Criteria for Adverse Event (CTCAE) v 4.0. The follow-up schedule for study visits is outlined in Table 1. Assessments at weeks 5 and 6 were required in the treatment arm only. Study assessment time points are further described in Table 2.

**Table 2:** Study assessments and time points

Study Assessments/Testing	Baseline	Day									
		-2	0	7	14	21	28	35	42	Next Clinic Visit	100
Demographics (patient-, disease-, and treatment-related)	X										
Additional descriptive outcomes				X	X	X	X				X
Socioeconomic status	X										
Hospital Anxiety and Depression Scale (HADS)	X		X	X	X	X	X				
Blood draw for gene expression analysis	X	X					X				
Pregnancy test for females of child bearing potential	X										
Toxicity			X	X	X	X	X	X^a^	X^a^		
Assessment of adherence^b^		X					X			X	
Heart rate and blood pressure	X		X	X	X	X	X			X	
Myeloma response assessment											X

^a^Propranolol group only

^b^Adherence assessed weekly or monthly based on number of pills prescribed

Participants who needed to stop propranolol therapy secondary to intolerance or new onset of a contraindication were not considered for resumption of therapy. Outcome and medical data continued to be collected and assessed for intervention arm participants despite an inability to remain on ß-blocker therapy for the study duration.

### Data collection and management

#### Specimen collection

A CRC drew blood at three study time points as described in Table 2. These time points included baseline (Day − 7; Time 1), Day − 2 (immediately prior to transplant, central line placement, or administration of any conditioning regimen; Time 2), and Day + 28 (Time 3). Blood was drawn in the hospital or transplant clinic depending on patient location.

#### Outcome measures

In this paper, we report on the clinical feasibility of utilizing the ß-blocker propranolol in multiple myeloma patients undergoing first autologous HCT in this proof-of-concept pilot RCT. We assess feasibility by enrollment rate, tolerability (as assessed by adherence and side effect profiles), adherence, and retention rates. An estimate of 55–70% enrollment was proposed to indicate feasibility based on other biobehavioral oncology trials [51]. Only one prior study has formally assessed ß-blocker tolerability (in a cardiac population) and used the Short Form (36) Health Survey (SF-36) [52]. This tool was deemed too non-specific to ß-blocker therapy in particular to be useful in ascribing any potential side effects during the transplant process to ß-blocker usage. Therefore, a comprehensive clinical medical assessment by the transplant team to evaluate any side effects related to propranolol was used*.* Tolerability was assessed according to the National Cancer Institute’s CTCAE v 4.0. All grade 3–5 AE’s were collected for the entire cohort, including any serious AE’s. Propranolol was deemed tolerable if > 80% of the patients could remain on the drug (at either 20 mg or 40 mg bid) without experiencing side effects requiring drug cessation and did not experience any serious AE’s.

Adherence with study medication was assessed through a final pill count by the CRC at the end of the study, a method often used in psychotropic drug studies where drug metabolite levels are not routinely available, as with propranolol. Pill count represents a conservative estimate of drug adherence [53]. Drug adherence was measured as a percentage of the prescribed number of pills that were actually taken (either 20 mg or 40 mg bid)*.* There is no consensual standard for what constitutes adequate adherence, though some clinical trials define rates above 80% as acceptable [54]. Therefore, study participants were considered adherent if they took at least 80% of administered pills. Finally, a 70% retention rate was posited as deeming feasibility based on this being the average retention rate across clinical trials (https://forteresearch.com/news/infographic-retention-in-clinical-trials-keeping-patients-on-protocols/). Retention was defined as the percentage of patients to successfully continue with study participation whom did not voluntarily drop out and were able to be followed up on.

The study’s primary outcome was gene expression as a function of propranolol administration (summarized below in Primary analyses section). Expression levels of ß-adrenergic mediated gene expression will be compared between individuals randomized to propranolol vs. control just prior to HCT as well as 28 days following autologous HCT for multiple myeloma. Quantification of whole genome RNA production will be performed with specific identification of expression of ß-adrenergic signaling pathways and a priori-specified pathways involved in inflammation and antiviral responses (see below). All gene expression profiling assays will be conducted by personnel blind to sample identity and study conditions using automated sample processing and analysis protocols.

### Analytic plan

#### Sample size and power

The current sample size estimate of 25 (12–13 per arm) was chosen based on previous studies with similar or smaller sample sizes that have evaluated gene expression as a function of psychosocial factors and yielded hundreds of differentially expressed genes that generate statistically significant results in higher-order bioinformatics [55, 56]. More specific sample size estimates are difficult to project with any greater accuracy, as there were no data on the effect size for gene expression differences as a function of propranolol in humans at the time of study design. To this end, the current study will inform future sample size estimations by generating effect size estimates and 95% confidence intervals to plan future studies investigating the repurposing of ß-blockers for antineoplastic purposes.

#### Primary analyses

The final outcome for the gene expression analysis will be expression levels of the group of genes comprising the conserved transcriptional response to adversity (CTRA) profile (data not presented here). The CTRA profile represents a systemic shift in gene expression of 53 indicator genes involved in up-regulated expression of pro-inflammatory genes and down-regulated expression of genes involved in type I interferon (IFN) responses and antibody synthesis [57–60] among circulating immune cells during extended periods of stress, threat, or uncertainty, consistent with the physiology of stress-associated illness [38, 55, 57, 58, 61, 62]. The University of California Los Angeles (UCLA) Social Genomics Core will provide genetic and statistical analyses for gene expression data.

#### Secondary feasibility analyses

As described above in the Outcome measures section, feasibility will be determined by enrollment rate (55–70%), tolerability (> 80% of patients able to remain on drug and/or no serious AE’s), adherence (> 80%), and retention rates (> 70%). Enrollment was calculated as percent of total patients screened that were effectively randomized to the trial. Tolerability was assessed according to the National Cancer Institute’s CTCAE v 4.0. All grade 3–5 AE’s were collected for the entire cohort, including any serious AE’s. Adherence was calculated at study completion on an individual level as the percentage of the total number of pills the patient was instructed to take that were actually taken, as assessed by bottle pill count. Retention was calculated as the percentage of total patients who successfully continued study participation, i.e. those who did not voluntarily drop out or complete follow-up.

## Results

**Fig. 2:**
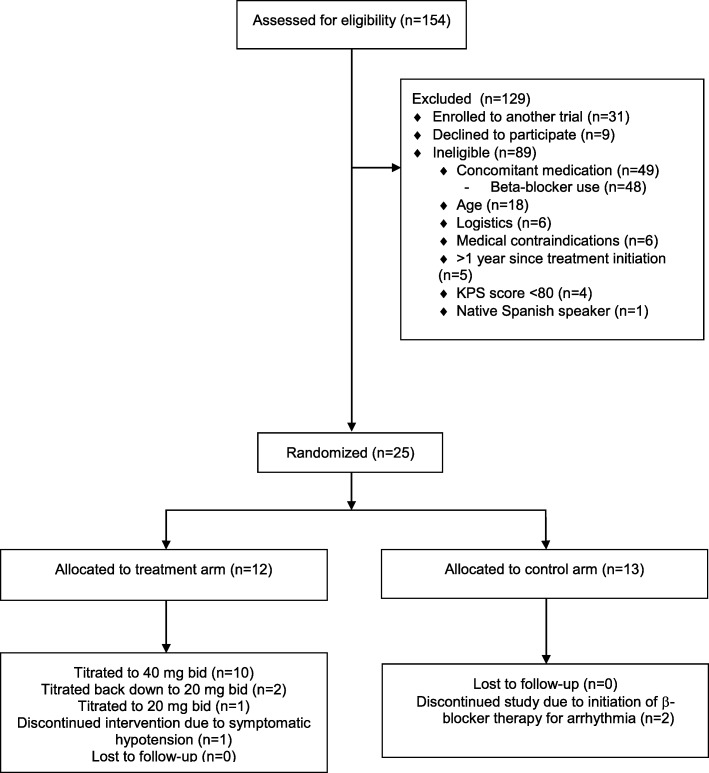
CONSORT Flow Diagram

Between July 2015 and March 2017, 154 patients were identified as meeting initial criteria of having a planned first autologous transplant for multiple myeloma*.* Comprehensive screening, eligibility, randomization, and exclusion criteria details are contained in Fig. 2. Nine patients declined study participation (reasons not systematically reported) and 31 were enrolled in other oncology treatment trials that did not permit co-enrollment. Twenty-five participants were enrolled. The most common reasons for ineligibility were current ß-blocker use (*n* = 48, 31%), age > 75 (*n* = 18, 12%), logistics (*n* = 6, 4%), medical contraindications (*n* = 6, 4%), myeloma treatment ≤1 year prior (*n* = 5, 3%), and KPS < 80% (*n* = 4, 3%). Medical contraindications included cardiac arrhythmias (*n* = 2), cardiomyopathy (*n* = 2), active depression (*n* = 1), pelvic fracture (*n* = 1), and Parkinson’s disease (*n* = 1). Thirteen patients were randomized to the control arm and 12 to the treatment arm. Overall enrollment rate was 16%.

No patients in either study arm experienced any serious AE’s. Of the 13 patients in the control arm, two patients came off study due to starting a ß-blocker post-transplant - one for tachycardia (Day + 18) and one for atrial fibrillation with rapid ventricular response (Day + 0). The other 11 control patients remained in the study for its entirety. The most frequent AE among the control group was hypertension, with 6 of the 13 control patients experiencing grade 3–5 hypertension. Three patients experienced hyperglycemia, with the following AE’s occurring two or fewer times: diarrhea, rash, cough, back pain, mucositis, abdominal pain, febrile neutropenia, spinal fracture, and periorbital infection.

Of the 12 patients in the treatment arm, 8 patients were successfully maintained at 40 mg bid without attributable adverse events (67%), 2 patients were titrated up to 40 mg bid but had to be reduced back down to 20 mg bid (17%), 1 patient remained at 20 mg bid due to preference (8%), and 1 patient was taken off study due to symptomatic hypotension (8%). Of the four patients who were not maintained at 40 mg bid, one patient was unable to complete the full course of propranolol due to persistent symptomatic hypotension as indicated by a blood pressure < 90/55 (Day + 4). This patient received propranolol 40 mg bid and had hypotension during a routine vital sign check. Drug dose was titrated down to 20 mg bid, but the patient remained hypotensive in subsequent evaluations and was taken off the study on day + 18 following transplant. A second patient experienced hypotension and dizziness at 40 mg bid, was titrated down to 20 mg bid, and was successfully continued on this dose without further symptomatology for the remainder of the study. A third patient remained at 20 mg bid due to fear of potential hypotension as she had had in the past, preceding the current study. This patient had no hypotensive episodes while on study. A fourth patient received 40 mg bid propranolol for the full course but experienced hypotension < 90/55 secondary to transplant-related diarrhea. The patient was treated with intravenous fluids, regained normal blood pressure, and was therefore continued on the full dose of propranolol. Other one-time adverse events among patients receiving propranolol included maculopapular rash, hypokalemia, hypertension, and chest pain, all of which were deemed unrelated or unattributable to the study intervention based on clinical assessment by the transplant team.

In sum, 11 of 12 (92%) patients were able to remain on the study drug. Four patients in the treatment arm had intervention-related hypotension (33%), only one of which was taken off of the study for this reason (8%). Two patients did not return their pill bottles to be assessed for final adherence; of the remaining 10 patients, 9 (90%) were adherent with the prescribed dosing regimen at the established > 80% cutoff. The remaining patient was adherent at a rate of 74%. Of patients who returned their pill bottles, the average proportion of pills taken was 94%. 100% of study participants were retained for evaluation of the primary endpoint.

## Discussion

Findings from this proof-of-concept RCT demonstrate that using prophylactic ß-blocker therapy with propranolol during HCT, a rigorous conventional cancer treatment regimen, is feasible as assessed by tolerability, compliance, and participant retention. Enrollment was lower than initially projected; however, in a busy multiple myeloma clinic at an academic medical center, with several competing trials open for enrollment concurrently, this study was successful in attaining our enrollment goal and we were able to enroll 16% of patients screened. These results demonstrate the feasibility of investigating the repurposing of existing drugs, here a ß-blocker, in a traditional oncology care/research setting, while also providing important information regarding the obstacle of enrollment competition with other oncology trials involving new immunotherapy agents. Conducting prospective patient trials of existing drugs for purposes of both treatment optimization [5] and containment of oncology care costs [1] is a critical component of improving cancer care and outcomes. This is particularly warranted for drugs that demonstrate anti-cancer efficacy in animal models [12–14] and are associated with improved outcomes in retrospective human studies [15–20].

The greatest feasibility challenge was enrollment. Our enrollment rate was lower than projected, in large part secondary to patients choosing other competing oncology trials involving new molecularly-targeted chemotherapeutic agents that did not allow for co-enrollment in other intervention trials. This important point should inform other trials that seek to evaluate repurposed drugs in cancer. We hypothesize this loss of potential participants may have been due to the perception for both patients and providers that repurposed drugs are not “active treatment”. Newer, more expensive immunotherapeutic agents may convey a strong sense of a promise for a “cure”, and as such be more enticing to both patients and providers. It would be interesting to know why patients chose to participate in the propranolol trial, sometimes at the exclusion of other newer immunotherapy trials. While patients volunteered reasons such as “I am anxious so this drug might help”, routine data collection regarding reasons to decline vs. enroll in a particular study contained insufficient detail to comment further. To improve our understanding of patient decision-making, there may be value in obtaining more detailed information in future studies of drug repurposing. While patients and providers may choose alternative studies, it is only ethical to offer patients all study options for which they are eligible. Despite this obstacle, the current trial was able to successfully enroll in a reasonable amount of time and meet our accrual target, highlighting that all potential participants were not lost to other drug studies. Our experience emphasizes the role for purposeful planning and enrollment algorithms to account for competing trials as well as the need for future study planning to anticipate competition with other immune-based oncology treatments, as they no doubt will continue to emerge. Nevertheless, the current study enrolled at a rate competitive with and exceeding that of other national oncology trials of cancer drugs at similar US academic medical centers; greater than 60% of these trials accrue fewer than 5 patients [63]. Finally, our enrollment rate was double that of another proof-of-concept prophylactic ß-blocker trial in a traumatically injured population [64].

Another less malleable factor in our lower-than-anticipated enrollment rate was the number of potential participants already on ß-blocker therapy (*n* = 48, 31% of screened participants). This percentage of patients already on therapy was comparable to that of another retrospective study evaluating multiple myeloma patients on ß-blockers (30.6%) [20]. While this factor is not modifiable, it should inform future prospective study planning and enrollment timelines for other ß-blocker cancer trials in a similarly-aged population.

Finally, 18 patients were ineligible owing to age > 75 years. Autologous HCT is a safe and effective treatment in older adults with myeloma. Over the last decade, the number of transplants in older adults for myeloma has considerably increased [31]. There is accumulating data on safety of transplants and equivalent outcomes to younger patients among older adults undergoing transplant for myeloma - even over 75 years of age - including data from our own center [65]. Thus for future similar studies, it would be justified to not have an upper age limit, and this would allow for more patients to be eligible for enrollment.

HCT patients are a high-risk study population on complicated medication regimens and with prolonged periods of immunosuppression and isolation [32]; the probability of 5-year survival after HCT is 15–55% depending on pre-transplant disease status and transplant and donor type [31]. Therefore, there is significant potential for drug side effects and lack of tolerability. In this study, propranolol was deemed tolerable if the patient could remain on the drug without experiencing side effects that required drug cessation. The study target was for > 80% of patients able to remain on drug and/or experience no serious AE’s. Side effects of hypotension and dizziness were not unexpected in the propranolol group [47]; hypotension was the most common intervention-related adverse event, with three patients in the treatment arm experiencing an episode (25%), and only one discontinued from the study for such. A notable difference between the control vs. treatment groups was the incidence of grade 3–5 hypertension, with six control individuals experiencing a grade 3–5 hypertension episode vs. only one in the treatment group. This suggests that while there may be more hypotensive events among patients on propranolol, this may be countered by the benefit in prophylactically alleviating significant hypertensive episodes.

In the recently published prospective trial of propranolol (along with cyclooxygenase-2) in early-stage breast cancer patients prior to surgery, one treatment arm patient experienced nausea as a side effect (5% of the treatment arm population) and subsequently withdrew from the study; there were no reported episodes of hypotension, though the continued propranolol dose was low at 20 mg bid [28]. In another recently published trial of perioperative propranolol for ovarian cancer patients, several doses of propranolol among the ten patients in the treatment arm were skipped due to nausea, though there were no episodes of hypotension [29]. It is important to consider the marked differences between these oncology populations and ours. Patients undergoing HCT for multiple myeloma are significantly more ill and receive chemotherapy (melphalan), resulting in loss of appetite, immune suppression, and infection susceptibility. In contrast, patients in both of those studies had not received any treatment at the time of enrollment, and apart from a breast or ovarian cancer diagnosis were not medically ill. Further, both of those studies did not administer propranolol for as many days as the present study; it is unknown which administration modalities may be most effective and in what context. Nonetheless, despite the increased vulnerability of our study population and occurrence of hypotensive events, the majority of patients were able to remain on the study drug without serious sequelae.

This study demonstrated an average 94% adherence rate among patients who returned their pill bottles. This rate substantially exceeds the posited rate of 80% to establish drug adherence in clinical trials [54] and thus the current study was deemed feasible with respect to adherence. Of note, two patients did not return their pill bottles for adequate count, so it is possible these patients were not adherent. However, there is no data about these patients to suggest they would have been different. In a recent study of adherence to HCT treatment regimens, patients reported adherence of approximately 66% to oral medications, including their non-investigational, standard immunosuppressant medications [66]. The adherence rate for the current study was significantly higher than in that study. Notably, just less than half of the medication administration time occurred while patients were inpatient and administered medications by a nurse, suggesting patients were adherent to their medication in the outpatient setting as well.

While recruitment is an important issue in determining feasibility of a clinical trial, participant retention is equally important in ensuring study success [67]. We were successful in retaining patients in this clinical trial, with a 100% retention rate. As such, we exceeded our goal retention rate of 70% and the 70% average retention rate across clinical trials (https://forteresearch.com/news/infographic-retention-in-clinical-trials-keeping-patients-on-protocols/). This may be in part related to the high level of engagement traditionally present among HCT recipients.

The study’s strengths include its successful implementation in a cancer population undergoing complicated medical procedures, and 100% retention rate. Treating physicians were sufficiently engaged and referred patients for study participation. Blood samples were successfully drawn and self-report surveys effectively administered in coordination with the complex treatment schedule of HCT. The study’s findings are limited in several respects. First, this is a relatively small sample size at a single institution, and study arms were not blinded. However, the sample size is not markedly different from that of other proof-of-concept studies of ß-blockers in non-oncology populations [64] or of other non-chemotherapy drug interventions in HCT populations that reported significant clinical outcomes [68]. Additional single and multi-site studies are needed to determine the generalizability of our findings. Next, our study is limited to one cancer treatment. Nevertheless, its demonstrated feasibility is significant given the complexities, both medically and logistically, of HCT as compared to other traditional oncology treatments in terms of enrolling and retaining patients on drug trials. The next step is to evaluate the efficacy of this repurposed drug in reducing neoplastic processes and improving clinical outcomes. Based upon our prior work indicating that the CTRA gene expression profile and/or its related transcriptome dynamics are significantly associated with relapse and progression-free survival [38, 69], these clinical outcomes may be worth evaluating in larger future clinical trials of propranolol.

## Conclusions

We have detailed the significance, rationale, study design, methodology, and feasibility of utilizing a repurposed drug – in this case the ß-blocker propranolol - with the rigorous conventional cancer treatment regimen of HCT for individuals with multiple myeloma. The study successfully recruited and retained its target sample size and demonstrated feasibility of administering a ß-blocker as a potential adjunctive antineoplastic medication during cancer treatment. Importantly, the current study enrolled at a similar rate and retained patients at a greater rate compared to other local and national oncology studies, contributing to evidence supporting the feasibility of implementing clinical trials of repurposed drugs in the clinical oncology setting. These data should support further studies that examine propranolol or other potentially repurposed drugs in other oncology populations.

## Abbreviations

AE: Adverse event
bid: Twice daily
CRC: Clinical Research Coordinator
CTCAE: Common Terminology for Adverse Event
CTRA: Conserved transcriptional response to adversity
HCT: Hematopoietic cell transplantation
HED: Human equivalent dose
IFN: Type I interferon
IRB: Institutional Review Board
KPS: Karnofsky Performance Score
MCW: Medical College of Wisconsin
PI: Principle Investigator
RCT: Randomized controlled trial
ßAR: ß-adrenergic receptor
ß-blocker: ß-adrenergic antagonist
UCLA: University of California Los Angeles
